# Whole genomic analysis uncovers high genetic diversity of rifampicin-resistant *Mycobacterium tuberculosis* strains in Botswana

**DOI:** 10.3389/fmicb.2025.1535160

**Published:** 2025-02-11

**Authors:** Tuelo Mogashoa, Johannes Loubser, Ontlametse T. Choga, Justice Tresor Ngom, Wonderful T. Choga, Mpaphi B. Mbulawa, Tuduetso Molefi, One Stephen, Topo Makhondo, Kedumetse Seru, Patience Motshosi, Boitumelo Zuze, Joseph Makhema, Rosemary M. Musonda, Dimpho Otukile, Chawangwa Modongo, Botshelo T. Kgwaadira, Keabetswe Fane, Simani Gaseitsiwe, Rob M. Warren, Sikhulile Moyo, Anzaan Dippenaar, Elizabeth M. Streicher

**Affiliations:** ^1^Botswana Harvard Health Partnership, Gaborone, Botswana; ^2^South African Medical Research Council Centre for Tuberculosis Research, Division of Molecular Biology and Human Genetics, Faculty of Medicine and Health Sciences, Stellenbosch University, Cape Town, South Africa; ^3^Department of Medical Sciences, University of Botswana, Gaborone, Botswana; ^4^Botswana National Tuberculosis Reference Laboratory, Gaborone, Botswana; ^5^Botswana National Tuberculosis Program, Ministry of Health, Gaborone, Botswana; ^6^Victus Global Botswana Organization, Gaborone, Botswana; ^7^TB/HIV Program, Botswana-University of Maryland School of Medicine, Health Initiative (BUMMHI), Gaborone, Botswana; ^8^Department of Pathology, Division of Medical Virology, Faculty of Medicine and Health Sciences, Stellenbosch University, Cape Town, South Africa; ^9^School of Health Systems and Public Health, University of Pretoria, Pretoria, South Africa; ^10^Department of Immunology and Infectious Diseases, Harvard T.H. Chan School of Public Health, Boston, MA, United States; ^11^Family Medicine and Population Health, University of Antwerp, Antwerp, Belgium

**Keywords:** tuberculosis, Botswana, genetic diversity, rifampicin resistance, whole genome sequencing

## Abstract

**Background:**

The emergence of drug-resistant *Mycobacterium tuberculosis* (*M. tb*) strains remains a threat to tuberculosis (TB) prevention and care. Understanding the drug resistance profiles of circulating strains is crucial for effective TB control. This study aimed to describe the genetic diversity of rifampicin-resistant *M. tb* strains circulating in Botswana using whole genome sequencing (WGS).

**Methods:**

This study included 202 stored *M. tb* isolates from people diagnosed with rifampicin-resistant TB (RR-TB) between January 2016 and June 2023. Genomic DNA was extracted using the cetyltrimethylammonium bromide (CTAB) method. Library preparation was performed using the Illumina DNA prep kit following the manufacturer's instructions. Sequencing was done on Illumina NextSeq2000. TBProfiler software was used to identify known *M. tb* lineages and drug resistance profiles. Statistical analyses were performed on STATA version 18.

**Results:**

WGS analysis revealed multidrug resistance (57.9%: 95% CI; 50.7–64.8), Pre-XDR (16.8%, 95% CI: 11.9–22.7), RR-TB (20.2%: 95% CI: 14.98–26.5), and HR-TB (0.5%, 95% CI; 0.01–2.7). We identified a high genetic diversity with three predominant lineages: lineage 4 (60.9%, 95% CI; 53.8–67.7), lineage 1 (22.8%: 95% CI; 17.2–29.2), and lineage 2 (13.9%, 95% CI: 9.4–19.4). The most frequently observed drug resistance mutations for rifampicin, isoniazid, ethambutol, streptomycin, pyrazinamide, and fluoroquinolones were *rpoB* S450L (28.6%), *katG* S315T (60.5%), *embA*_c.-29_-28delCT, *embB* Q497R (31.7%), *rrs*_n.517C>T (47.1%), *pncA*_c.375_389delCGATGAGGTCGATGT (36.0%) and *gyrA* A90V (79.4%), respectively. No bedaquiline and delamanid resistance-associated mutations were detected.

**Conclusions:**

This study highlights the high genetic diversity of *M. tb* strains, with a predominance of lineage 4 among people with RR-TB in Botswana. It provides valuable insights into the genetic diversity of rifampicin-resistant *M. tb* strains circulating in Botswana.

## 1 Introduction

The emergence of drug-resistant *Mycobacterium tuberculosis (M. tb)* strains remains a threat to global tuberculosis (TB) control. Rifampicin (RIF) is a potent first-line drug for TB treatment that inhibits protein synthesis by binding to the β-subunit of the bacterial DNA-dependent RNA polymerase enzyme (*rpo*B) protein (Ramaswamy and Musser, 1998). Rifampicin resistance (RR) is defined as any resistance to RIF, and this includes RIF monoresistance, multidrug resistance (MDR) resistance to both isoniazid (INH and RIF) and resistance to RIF and any other drug (polydrug resistance) (Ogwang et al., 2021). Mutations in the *rpo*B gene, which codes for the β-subunit of the bacterial DNA-dependent RNA polymerase, have been shown to lead to RR (Salaam-Dreyer et al., 2021; Khosravi et al., 2012). The incidence of RR-TB continues to increase globally. The World Health Organization (WHO) estimated that there were 410,000 RR-TB cases in 2022 (WHO, 2023a). The current WHO recommendations for the treatment of RR-TB include the newer drugs bedaquiline (BDQ), pretomanid (PTM), delamanid (DLM), linezolid (LZD) and moxifloxacin (MOX) (Fong, 2023; World Health Organization, 2022).

Drug-resistant TB (DR-TB) is becoming an emerging threat to human health in Botswana; there has been a 3.1-fold increase in RR-TB cases since the last drug resistance survey, which was conducted between 2007 and 2008 by Menzies et al. (2014). Despite this increase in RR-TB, data on the notifications of RR-TB in Botswana remains limited. Hence, there is an urgent need for increased surveillance of RR-TB and associated additional drug resistance.

Moreover, the burden of the Tuberculosis/Human immunodeficiency virus (TB/HIV) co-infection rate in Botswana is ~60% (MOH, 2016), and the country is on the WHO list of countries with a high burden of HIV-associated TB (WHO, 2021).

Early detection of TB, as well as accurate and comprehensive drug susceptibility testing (DST), is critical for optimal TB treatment and to reduce the risk of further DR-TB development. Currently, culture-based phenotypic drug susceptibility testing (pDST) and rapid line probe assays (LPA) are some of the WHO-endorsed methods for identifying resistance to anti-TB drugs (WHO, 2018). The culture-based pDST methods are labor-intensive, require sophisticated laboratory infrastructure and biosafety measures, and may take more than 1 month for results to be reported, a period where additional resistance may be acquired (Jacobson et al., 2017). We previously described the genetic diversity of *M. tb* isolates in Botswana using spoligotyping, which has an inferior discriminatory power compared to the whole genome sequencing (WGS) (Mogashoa et al., 2019). WGS offers a rapid alternative to pDST by identifying causal mutations in all genes known to cause resistance (Cohen et al., 2019). WGS analysis can comprehensively predict drug resistance profiles of *M. tb* and can be used to identify and characterize transmission clusters (Dookie et al., 2018; Witney et al., 2015).

Molecular diagnostics generally only consider a limited number of targets, which may result in missing resistance-causing variants outside the canonical areas. For example, the Cepheid Xpert MTB/RIF and MTB/RIF Ultra assays (Cepheid, Sunnyvale, CA, USA) detect rifampicin resistance-associated mutations in the RIF resistance determining region (RRDR) of the *rpo*B gene only without considering other potential resistance conferring-genes (Helb et al., 2010). In contrast, the Cepheid Xpert MTB/XDR expands resistance detection to include isoniazid, fluoroquinolones, ethionamide, and amikacin (Pillay et al., 2022; Centner et al., 2024). Similarly, the LPAs such as the GenoType MTBDR*plus* (Hain Lifescience, GmbH, Nehren, Germany), recommended by WHO and detects mutations in the *rpo*B gene for RIF resistance, *kat*G gene for INH resistance, and *inh*A promotor region for low-level INH resistance. The GenoType MTBDR*sl* (Hain Lifescience, GmbH, Nehren, Germany) assay further extends the detection of mutations in the *gyrA* and *rrs* genes, which are linked to resistance to fluoroquinolones and second-line injectable drugs, respectively (Rahman et al., 2021). However, these molecular diagnostics do not cover newer drugs and developing rapid molecular diagnostics for anti-TB drugs with multiple gene targets, such as BDQ and DLM, may be cumbersome (Machado et al., 2019). In this regard, WGS offers a potential “all-in-one” comprehensive solution for DR profiling, strain characterization, and transmission analysis. In this study, we aimed to describe the genetic diversity and drug resistance profiles of rifampicin-resistant *M. tb* strains circulating in Botswana using WGS.

## 2 Materials and methods

### 2.1 Study population

This was a retrospective cross-sectional study in which we characterized 202 archived *M. tb* isolates from the National Tuberculosis Reference Laboratory (NTRL), which were collected from people who were diagnosed with rifampicin-resistant tuberculosis (RR-TB) from TB clinics around the country between 1st January 2016 and 30th June 2023. During this period, ~300 people were diagnosed with MDR/RR-TB and we included one *M. tb* isolate per participant in our analysis.

### 2.2 Phenotypic drug susceptibility testing test

Presumptive TB cases were first screened with Cepheid Xpert MTB/RIF or MTB/RIF Ultra assays (Cepheid, Sunnyvale, CA, USA), and after *M. tb* was detected phenotypic drug susceptibility test (pDST) was performed at the NTRL with the fluorometric method BACTEC Mycobacterium Growth Indicator Tube (MGIT) 960 (Becton-Dickinson, Franklin Lakes, NJ USA) for the five first-line antibiotics: isoniazid (INH), rifampicin (RIF), ethambutol (EMB), ethionamide (ETH), streptomycin (STR) and pyrazinamide (PZA). The critical concentrations used in the DST analysis were INH = 0.1 μg/ml, RIF = 1.0 μg/ml, EMB = 5.0 μg/ml, ETH = 5.0 μg/ml and STR = 1.0 μg/ml. Pyrazinamide DST (100 μg/ml) was performed using the BACTEC MGIT 960 PZA kit (Becton Dickinson, Franklin Lakes, NJ, USA). Second line DST was not performed.

### 2.3 Genomic DNA extraction

The genomic DNA of *M. tb* isolates was extracted using the Hexadecyltrimethylammonium bromide (CTAB) method as previously described (van Embden et al., 1993). Briefly, the biomass from 2 months of growth in Lowenstein-Jensen tubes was resuspended in 400 μl of 1X TE buffer. Bacterial cells were heat-killed by incubating the cell suspension at 80°C for 20 min, followed by overnight incubation at 37^o^C with 50 μl of 10 mg/ml lysozyme solution (Glentham Lifesciences, Planegg, Germany). Cellular lysis was performed by adding 75 μl of 10% SDS/proteinase K mix (5 μl of 10 mg/ml Proteinase-K (New England Biolabs, USA) and 70 μl 10% SDS), then mixing and incubating at 65°C for 10 min. Then, 100 μl of 5M NaCl and 100 μl of pre-warmed (65°C) solution of CTAB/NaCl (40 mM/0.1M) were added, and the suspension was mixed vigorously by vortexing, and incubated at 65°C for 10 min. DNA extraction was achieved by adding an 750 μl of chloroform-isoamyl alcohol (24:1) (Glentham Lifesciences, Planegg, Germany) to the lysate, which was then mixed and centrifuged for 15 min at 13,000 g at room temperature. The aqueous phase was treated with 0.6 volumes of isopropanol to precipitate the DNA. The resulting DNA pellet was then washed twice with 500 μl of cold 70% ethanol solution. The pellet was air dried at 60°C for 20 min, and the DNA was resuspended in 50 μl AE buffer (5 nM 10 mM Tis/HCl, pH 8.5). The DNA was dissolved by incubating them at 60°C for 20 min. DNA integrity was verified by electrophoresis in a 2% agarose gel (Thistle Scientific, Warwickshire, UK). DNA concentration and purity were determined by spectrometry with a Nanodrop 2000 (ThermoScientific, Waltham, Massachusetts, USA) and DNA was quantified by fluorometry with a Qubit v.3 using the Qubit dsDNA HS Assay Kit (Invitrogen, Waltham, Massachusetts, USA).

### 2.4 Whole genome sequencing and bioinformatics analyses

Genomic libraries were prepared from 100 ng high-quality genomic DNA using the Illumina DNA Prep kit (Illumina, San Diego, CA, USA), following the manufacturer's instructions. Samples that passed the quality control were pooled and normalized according to the manufacturer's recommendations. The pooled library was loaded into a 300-cycle P2 cartridge at 2 pM. Sequencing was performed using the Illumina NextSeq2000 sequencer (Illumina, San Diego, C.A., USA) in a 2 × 151 bp paired-end format.

FASTQC (http://www.bioinformactics.babraham.ac.uk/projects/fastqc/) was used as a primary data assessment to check the quality of sequence reads. Taxonomic classification was conducted with the Kraken2 tool version 2.1.3 (Wood et al., 2019). Read alignment and variant calling were performed using the MTBseq pipeline version 1.1.0 (Kohl et al., 2018). The MTBseq analysis provided the mapping statistics, the sub-lineage classification, the identification of transmission groups and a list of amended variants. Quality control of aligned reads was done using Qualimap version 2.2.2 software (Okonechnikov et al., 2016). The criteria for sequencing data quality were mean depth of coverage ≥30 × and mapped reads percentage ≥95%. Drug resistance genotypes for both first- and second-line antibiotics were determined using TB-profiler software, version 4.4.2 (Phelan et al., 2019).

### 2.5 Phylogenetic analyses

A phylogenetic tree was reconstructed using a list of concatenated SNPs derived from the MTBseq pipeline, applying the default parameter to define the transmission groups. To generate a maximum-likelihood tree supported by bootstrap values, a tree was inferred from the FASTA file using IQ-TREE (Minh et al., 2020) with -m MFP option to choose and utilize the best fitting model of nucleotide substitution. The best fitting model was TVM+F+R3. A total of 1,000 bootstrap replicates was used to infer support for branches in the resulting tree; bootstrap >80% were considered significant. Annotation and visualization of the phylogenetic tree was conducted with the online software tool, Interactive Tree of Life (iTOL) version 6.8.1) (Letunic and Bork, 2021).

### 2.6 Statistical analysis

Baseline demographics were summarized using descriptive statistics. The continuous variables were reported as medians with first (Q1) and third (Q3) quartiles. Proportions of drug resistance mutations were estimated within individuals with resistance toward first line and second-line TB drugs. The proportions were further stratified by HIV status and compared using a comparison of proportions test. The association of specific drug resistance mutations with TB lineages were assessed using chi-square test or Fishers' exact test. All the *p*-values ≤ 0.05 were considered statistically significant. All the data analyses were performed using STATA version 18.

## 3 Results

In this study, WGS was successfully performed for 202 rifampicin-resistant *M. tb* isolates collected between January 2016 and June 2023. Demographic characteristics are shown in Table 1. The median age of participants was 37 (Q_1_, Q_3_: 28, 48) and most of the participants were male (*n* = 128, 63.4%). Most of the study population were newly diagnosed TB cases (*n* = 177, 87.6%), while 49% (*n* = 99) were people living with HIV.

**Table 1:** Demographic characteristics of patients with rifampicin-resistant tuberculosis (*n* = 202).

**Characteristic**	***N***	**Percentage**
**Sex (*****n*** = **202)**		
Male	128	63.4
Female	74	36.6
Median age in years (Q1, Q3) 37 (28, 48)		
**HIV status**		
Positive	99	49.0
Negative	76	37.6
Unknown	27	13.4
**Site of TB**		
Pulmonary	197	97.5
Extrapulmonary	5	2.5
**Smear result**		
Negative	43	21.9
Positive	159	78.1
**Treatment history**		
New cases	177	87.6
Previously treated	25	12.4

### 3.1 Sequencing data quality and lineage classification

The median sequencing depth, number of reads mapped, and percentage of reads mapped were 157 (Q_1_, Q_3_: 121, 272), 7.35 × 10^6^ (Q_1_, Q_3_: 5.08 × 10^6^, 1.50 × 10^7^) and 99.7 (Q_1_, Q_3_: 99.2, 99.5), respectively. Sequence data was used to predict resistance patterns and perform phylogenetic classification.

**Figure 1:**
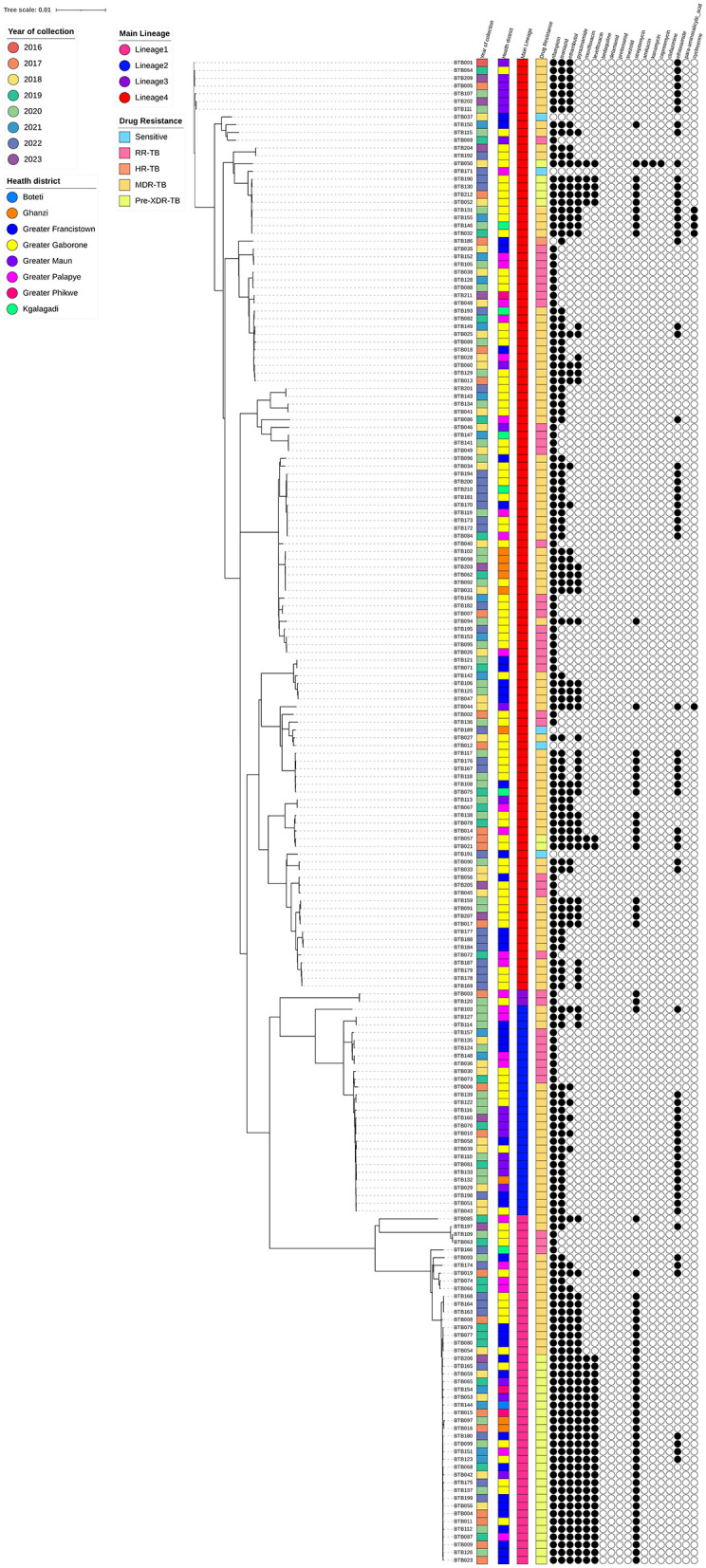
Maximum-likelihood phylogeny of 202 rifampicin-resistant *Mycobacterium tuberculosis* isolates. The color-coded annotations include year of collection, health district, main lineage and drug resistance profiles. The black and white circles indicate the genotypic resistance profiles to 18 antibiotics (black and white represent resistance and susceptibility to the specific antibiotics, respectively).

The *M. tb* strains were found to be genetically diverse, with most of them belonging to lineage 4 (*n* = 123, 60.9%), with the least prevalent lineage being lineage 3 (*n* = 2, 1%; Figure 1).

### 3.2 Drug resistance

#### 3.2.1 Phenotypic drug resistance

Phenotypic DST results were available for 194 of the 202 sequenced isolates. Table 2 shows the drug resistance profiles of the 194 *M. tb* isolates with phenotypic DST results for first-line drugs (RIF, INH, EMB, STR and PZA). Excluding the eight isolates without pDST results, there were RR (45, 23.2%) and MDR (149, 76.8%) isolates.

**Table 2:** Phenotypic drug resistance patterns of 194 *M. tb* isolates.

**Drug-resistant profiles**	**No. of isolates (%)**
RR	45 (23.2)
RIF only	31(16.0)
RIF + EMB	4 (2.1)
RIF + STR	10 (5.2)
MDR	149 (76.8)
RIF + INH	49 (25.3)
RIF + INH + EMB	20 (10.3)
RIF + INH + STR	14 (7.2)
RIF + INH + EMB + STR	66 (34.0)

RIF, rifampicin; INH, isoniazid; EMB, ethambutol; STR, streptomycin; RR, rifampicin resistance; MDR, multidrug resistance.

pDST was not done for eight isolates.

### 3.3 Genotypic resistance

The Supplementary Table 1 presents a comprehensive summary of the drug resistance mutations identified in this study, detailing the specific mutations, associated lineages, the number of isolates and the final WHO confidence grading (WHO, 2023b).

#### 3.3.1 Rifampicin resistance

A high diversity of *rpoB* mutations was observed. Among the 192 phenotypically RIF-resistant isolates, 56 (29.2%) had r*poB* S450L, and 36 (18.8%) had the *rpoB* H445L mutation which are both associated with high level resistance to RIF. Two isolates (1%) concurrently exhibited *rpoB* S450L and putative *rpoC* I491T mutation. Mutations outside the rifampicin resistance-determining region (RRDR) (I491F and I170V) were seen in 12 (6.3%) isolates. There were six (3.1%) isolates which did not harbor any mutations associated with rifampicin resistance.

#### 3.3.2 Isoniazid resistance

There was at least one known resistance-conferring variant in *katG* or *inhA*. Among the 150 phenotypically INH-resistant isolates, 90 (60%) had high-level INH resistance conferring mutation *katG* S315T, while 9 (6.0%) isolates had the *inhA* S94A mutation. There were 21 (14.0%) isolates with a combination of *inhA*−770 T>A + *katG* S315T; 5 (3.3%) isolates had a combination of *ahpC*−57C>T + *katG* S315T, 1(0.7%) isolate had a combination of *ahpC*−57C>T + *katG* S315T, 1 isolate (0.7%) had *inhA*−154G>A mutation, 5 isolates (3.3%) had *inhA*−154G>A, 4 (2.7%) *inhA*−777C>T, 5 (3.3%) *inhA*−777C>T + *inhA* I21T, 9 (6.0%) isolates did not harbor any INH resistance conferring mutations.

#### 3.3.3 Ethambutol resistance

Among the 89 phenotypically EMB-resistant isolates, 35 (39.3%) exhibited the *embA*−29_-28delCT + *embB* Q497R mutation, while 18 (20.2%) and 12 (13.4%) isolates had high confidence *embB* M306V and *embB* M306I mutations respectively (other mutations are detailed fully in Supplementary Table 1).

#### 3.3.4 Streptomycin resistance

Among the 90 phenotypically STR-resistant isolates, 35 (38.9%) had *rrs* 517 C>T, while 10 (11%) had *rpsL* K43R mutation which is associated with high level resistance to STR (other mutations are detailed fully in Supplementary Table 1).

#### 3.3.5 Ethionamide resistance

Phenotypic DST was only done for 34 isolates, and among these, 20 (58.8%) did not exhibit any drug resistance mutations, while 4 (11.8%) had the *inhA* S94A mutation. Two isolates had the *inhA*−777C>T + *inhA* I21T (5.9%) mutation, one isolate had *inhA*−777C>T mutation (2.9%) and 2 isolates had the *ethA* 860delA mutation (other mutations are detailed fully in Supplementary Table 1).

#### 3.3.6 Pyrazinamide resistance

Phenotypic DST was only done for 22 isolates; among these isolates, 12 (54.5%) did not exhibit any mutation, 4 (18.2%) isolates had the *pncA* 375_389delCGATGAGGTCGATGT mutation, two (9.1%) isolates had the *pncA* 391dupG mutation (other mutations are detailed fully in Supplementary Table 1).

### 3.4 *M. tb* lineages

The isolates in this study were genetically diverse and belonged to lineage 1 (46, 22.8%), lineage 2 (28, 13.9%), lineage 3 (2, 1%) and lineage 4 (123, 60.9%). In lineages 1, 2 and 4, the most predominant sub-lineages were 1.2.2.2 (42, 20.8%), 2.2.1 (27, 13.4%) and 4.4.1.1 (27, 13.4%), respectively. In addition, there were three cases (1.5%) of mixed lineages, with lineage 4 being common in all cases.

### 3.5 Association of specific drug resistance mutations with *M. tb* lineages

Table 3 presents data on the frequencies of specific drug resistance mutations across the three *Mtb* lineages. Mutation *rpoB* S450L (39%) was most common in lineage 4 strains, while *rpoB* H445L (67.4%) and *katG* S315T (82.6%) were more common in lineage 1 strains. Several mutations are strongly associated with specific lineages: *rpoB* H445L is predominantly found in lineage 1, while *gyrA* A90V only occurred in lineage 1 isolates, and *inhA*−770T>A is unique to lineage 2. The mutations *rpoB* S450L, *rpoB* H445L, *katG* S315T, *embB* M306V, and *inhA*−777C>T show significant differences in their distribution across the lineages (Table 3).

**Table 3:** Drug resistance mutations stratified by *M. tb* lineages in rifampicin resistant isolates (*n* = 197).

**Drugs/gene mutation**	**Lineage 1 (*n* = 46)**	**Lineage 2 (*n* = 28)**	**Lineage 4 (*n* = 123)**	***p* value**
	***n*** **(%)**	***n*** **(%)**	***n*** **(%)**	
RIF/*rpoB* S450L	2 (4.3)	8 (28.6)	48 (39)	0.000
RIF/*rpoB* H445D	3 (6.5)	1 (3.6)	17 (13.8)	0.17
RIF/*rpoB* H445L	31(67.4)	5 (17.9)	2 (1.6)	0.000
INH/*katG* S315T	38 (82.6)	21(75)	67 (54.5)	0.001
INH/*inhA* S94A	1 (2.2)	0	9 (7.3)	0.12
INH/*inhA*−777C>T	1 (2.2)	0	13 (10.6)	0.048
EMB/*embB* M306V	0	3 (10.7)	17 (13.8)	0.03
EMB/*embB* M306I	0	2 (7.1)	12 (9.8)	0.09
EMB/*embB* Q497R	2 (4.3)	1(3.6)	14 (11.4)	0.21
PZA/*pncA* G132A	0	0	8 (6.5)	N/A
PZA/*pncA* T135P	0	0	6 (4.9)	N/A
PZA/*pncA* G97C	0	0	5 (4.1)	N/A
FQs/*gyrA* A90V	27 (58.7)	0	0	N/A
FQs/*gyrA* D94A	0	0	2 (1.6)	N/A
FQs/*gyrA* D94G	0	0	4 (3.3)	N/A
ETH/*inhA* S94A	1 (2.2)	0	9 (7.3)	0.100
ETH/*inhA*-770T>A	0	16(57.1)	0	N/A
ETH/inhA-777 C>T	1 (2.2)	0	13 (10.6)	0.048

RIF, rifampicin; INH, isoniazid; EMB, ethambutol; PZA, pyrazinamide; FQs, fluroquinolones; KM, kanamycin; AMK, amikacin; CM, capreomycin; ETH, ethionamide; N/A, not applicable.

## 4 Discussion

This study provides a comprehensive genomic characterization of 202 *M. tb* isolates from TB clinics in Botswana using WGS. We focused on characterizing rifampicin-resistant isolates. Our findings show a high genetic diversity of rifampicin-resistant strains. Consistent with previous studies in Botswana (Mogashoa et al., 2019; Click et al., 2020), we found that lineage 4 was predominant in our study population, followed by lineage 1, and lineage 3 being the least common lineage. Botswana's overall strain diversity closely resembles that of other African and neighboring countries such as South Africa, Namibia and Zambia, where lineage 4 also accounts for the majority of circulating *M. tb* strains (Chisompola et al., 2021; Mbugi et al., 2016; Chihota et al., 2018; Claassens et al., 2022). The historical movement of people between these countries for commerce and trade has likely contributed to the spread and transmission of this lineage across Southern Africa (Crush et al., 2005).

We report a high diversity of *rpoB* mutations from epidemiologically linked and unlinked samples, potentially implying that the bacteria have undergone significant genetic changes over time on multiple occasions. This high diversity also suggests that the strains may have been potentially introduced from different sources (Carey et al., 2018; Gagneux, 2018; Stucki et al., 2016). In this study we identified *rpoB* mutations which are associated with both low- and high-level RIF-resistance. Previous studies have shown that strains with borderline or low-level resistance mutations can transmit at rates similar to those with common mutations associated with high-level resistance despite potential fitness costs (Lempens et al., 2023). As reported in previous studies, the most prevalent *rpoB* and *katG* mutations were S450L and S315T, respectively (Claassens et al., 2022; Traoré et al., 2023; Solo et al., 2020). Each *M. tb* lineage exhibited distinct patterns of drug resistance mutations. Lineage 1 strains predominantly had *rpoB* H445L and *katG* S315T mutations. In contrast, lineage 4 strains had a predominance of *rpoB* S450L, *inhA*−777 C>T and *embB* M306V, which suggests a statistically significant difference in the distribution of these mutations across the different lineages. From our findings, we show that each *M. tb* lineage exhibits a distinct pattern of drug resistance mutations which can potentially influence the effectiveness of TB treatment regimens in Botswana. Previous studies have shown that there is an association between specific drug resistance mutations and increased minimum inhibitory concentration (MIC) for different anti-TB drugs; mutations in the *rpoB* at codons 450 and 445 have been linked to increased MICs for RIF (Ruesen et al., 2018; Barilar et al., 2024). These findings further highlight the need to understand how strain genetic background and mutations impact MIC levels as this could provide a rapid and reliable alternative to pDST and improve TB diagnostics (Getahun et al., 2022). However, our study was limited by the unavailability MIC data which restricts more detailed analyses. The identified mutations in this study warrant future studies to further investigate their association with MIC.

Notably, we also identified resistance-conferring mutations outside the rifampicin resistance-determining region (RRDR) of the *rpoB* gene, i.e. *rpoB* I491F and I170V. These mutations are not detected by current rapid molecular diagnostic tools such as the GenoType MTBDR*plus* and may be overlooked by pDST. The presence of such mutations could potentially lead to misdiagnosis, underreporting of rifampicin resistance, and potentially inadequate treatment regimens (Takawira et al., 2017). Notably, a recent study reported a case where rifampicin resistance was undetected by the GeneXpert MTB/RIF assay in a participant enrolled in the study. It was later discovered through WGS that the participant indeed had RR-TB caused by a strain with the *rpoB* I491F mutation (Modongo et al., 2023). In this study, we report 8 strains that harbored the *rpoB* I491F mutation and belonged to sub-lineage 4.3.3, and these strains are similar to the strain that was recently reported by Modongo et al. (2023). Recent studies by Makhado et al. (2018) and Sanchez-Padilla et al. (2015) reported on *M. tb* strains with *rpoB* mutation I491F which were undetected by WHO-endorsed rapid molecular assays (Mvelase et al., 2023). Interestingly, the strains identified in eSwatini and South Africa belonged to lineage 4.4.1.1 and lineage 4.1.1.3, which differ from the strains detected in our study; this is suggestive of convergent evolution of *M. tb* strains with this mutation in Southern Africa. Mutations such as *rpoB* I491F may compromise the efficacy of RIF-based regimens, resulting in treatment failure and potentially increasing the risk of onward transmission. This underscores the complexity of TB transmission in the region and highlights the need for enhanced molecular surveillance to detect these resistant strains effectively.

Based on ML-tree phylogenetic tree inferences, the majority of the sequences belonged to Lineage 4 and were predominantly sampled from the greater Gaborone region, where the majority formed multiple small monophyletic clusters. Some of the multiple clusters were identified in different regions, suggesting possible local transmission. Even though, there was no consistent pattern observed in these clusters based on sampling year. Follow-up studies of phylogenetics and phylogeography are important to further investigate these clusters. In contrast to Lineage 4, other lineages (1, 2, and 3) exhibited mostly random clustering patterns, likely due to the limited sample sizes.

We identified six rifampicin resistant *M. tb* isolates, which did not harbor any mutations which are associated with RIF resistance. A recent study suggested that inactivation of a potassium channel may increase resistance to RIF, suggesting that this channel may play an important role in drug uptake (Do et al., 2022). Our study did not evaluate the inactivation of the potassium channel in these six isolates which we acknowledge as a limitation. This highlights the need for a more comprehensive analyses to investigate alternative resistance mechanisms that might explain RIF resistance in these six *M. tb* isolates without any canonical rifampicin resistance mutations. Another significant finding is the absence of mutations conferring resistance to newer and repurposed drugs, such as bedaquiline and delamanid. These drugs are part of the shorter regimens for treating MDR-TB. The lack of resistance to these drugs in our study is encouraging, as it suggests that these treatments remain effective in Botswana. Compared to the latest WHO catalog of mutations associated with drug resistance; we identified several mutations currently not in the WHO catalog of mutations (WHO, 2023b). These mutations require further investigation to assess their clinical relevance and their potential impact on the MIC of RIF, PZA and ETH.

### 4.1 Limitations

The limitation of this study is sampling bias, as we only included isolates which were available in the biorepository, which may not fully represent the diversity of *M. tb* strains circulating in the broader population. Second-line pDST was not performed therefore the resistance profiles are less comprehensive. Incorporating second-line pDST in future would provide a more comprehensive resistance profile which may inform TB control efforts more effectively.

## 5 Conclusions

In conclusion, this study highlights the high genetic diversity of rifampicin-resistant *M. tb* strains in Botswana, with each lineage exhibiting distinct resistance patterns. The presence of diagnostic escape mutations emphasizes the need to improve TB testing algorithms to incorporate WGS or targeted sequencing tests to rapidly detect these variants as well as other variants which are not detected in the well-defined drug resistance-determining regions. Additionally, scaling up both genotypic and phenotypic drug susceptibility testing for new and repurposed drugs is also crucial to monitor the emergence of resistance to these drugs, ensuring that these new treatment regimens remain effective. Addressing these will be key to advancing TB control efforts in Botswana and similar high-burden settings.

## Data Availability

The original contributions presented in the study are publicly available. This data can be found here: https://www.ebi.ac.uk/ena/browser/home, accession number PRJEB83872.
