# Supplementary material for: Whole genomic analysis uncovers high genetic diversity of rifampicin-resistant Mycobacterium tuberculosis strains in Botswana
Source: Front Microbiol. 2025 Feb 11;16:1535160. doi: 10.3389/fmicb.2025.1535160 (PMC11855114; doi:10.3389/fmicb.2025.1535160)
Supplement: Supplementary file 1 [file Table_1.docx]

**Supplementary table 1: Catalogue of drug resistance-associated mutations of the 202 *Mtb* isolates**

| **Drug** | **Mutation** | **WHO final confidence grading** | **Number of isolates** | **Lineages of isolates (n)** |
| --- | --- | --- | --- | --- |
| Rifampicin | *rpoB* 12931295dupCCA | *not in the WHO catalogue | 1 | L4.3.2.1 (1) |
|  | *rpoB* 3031305delGAC | *not in the WHO catalogue | 3 | L4.4.1.1 (3) |
|  | *rpoB* 13121314delAAC | *not in the WHO catalogue | 3 | L4.1.2 (3) |
|  | *rpoB* A286V+ *rpoB* S450L | Assoc w R | 2 | L2.2.1 (2) |
|  | *rpoB* D435A + *rpoB* D435V | Assoc w R | 1 | L2.2.1 (1) |
|  | *rpoB* D435Y + *rpoB* T444I | Assoc w R | 1 | L4.3.4.2.1 (1) |
|  | *rpoB* D435V | Assoc w R | 8 | L4.3.2.1 (4)  L4.3.4.1 (1)  L4.3.3 (1)  L2.2.1 (2) |
|  | *rpoB* Q432L | Assoc w R | 5 | L4.1.2 (2)  L4.1.1.3 (3) |
|  | *rpoB* Q432P | Assoc w R | 2 | L4.3.2.1 (2) |
|  | *rpoB* H445R | Assoc w R | 2 | L2.2.1 (2) |
|  | *rpoB* H445N + *rpoB* L452P | Assoc w R | 1 | L1.2.2.2 (1) |
|  | *rpoB* H445D | Assoc w R | 20 | L1.2.2.2 (3)  L2.2.1 (1)  L4.1.1.1 (5)  L4.1.1.3 (1)  L.4.1.2 (1)  L4.3.2.1 (3)  L4.4.1.1 (6) |
|  | *rpoB* H445D + *rpoB* H445Y | Assoc w R | 1 | L4.8.1 (1) |
|  | *rpoB* H445C | Assoc w R | 1 | L4.1.1.2; L4.1.1.3 (1) |
|  | *rpoB* H445L | Assoc w R | 38 | L1.2.2.2 (31)  L2.2.1 (5)  L4.9.1 (2) |
|  | *rpoB* H445S + *rpoB* H445T + *rpoB* K446Q | Assoc w R | 2 | L2.2.1 (2) |
|  | *rpoB* H445Y | Assoc w R | 15 | L1.2.2.2 (2)  L2.2.1 (5)  L4.1.2.1 (2)  L4.3.4.2.1 (1)  L4.4.1.1 (2)  L4.8 (3) |
|  | *rpoB* L430P | Assoc w R | 1 | L1.2.2.2 (1) |
|  | *rpoB* I491F | Assoc w R | 8 | L4.3.3 (8) |
|  | *rpoB* I491F + *rpoB* S450L | Assoc w R | 1 | L4.3.4.1 (1) |
|  | *rpoB* S441L | Assoc w R | 1 | L4.3.4.2 (1) |
|  | *rpoB* S450L | Assoc w R | 59 | L1.1.3.2 (1)  L2.2.1 (7)  L2.2.2 (1)  L3 (2)  L4.1.1.3 (12)  L4.1.2.1(8)  L4.3.2 (3)  L4.3.2.1 (5)  L4.3.4.1 (4)  L4.3.4.2.1 (1)  L4.4.1.1 (11)  L4.8 (1)  L4.9 (2) |
|  | *rpoB* S450L *+ rpoB* T400A | Assoc w R | 1 | L1.1.2 (1) |
|  | *rpoB* S450L + *rpoC* I491T | Assoc w R | 2 | L4.3.3 (2) |
|  | *rpoB* S450F | Assoc w R | 1 | L4.3.4.2.1 (1) |
|  | *rpoB* S450W | Assoc w R | 7 | L4.4.1.1 (7) |
|  | *rpoB* S450Y | Assoc w R- interim | 2 | L1.1.3.2 (2) |
|  | *rpoB* V170F | Assoc w R | 4 | L1.2.2.2 (3)  L4.1.1.3 (1) |
| Isoniazid | *ahpC* -54C>T + *inhA* -154G>A + *inhA* I21V | Assoc w R | 1 | L1.2.2.2 (1) |
|  | *ahpC* -57C>T + *katG* S315T | Assoc w R | 6 | L4.1.2.1 (6) |
|  | *inhA* c + -154G>A | Assoc w R | 2 | L1.1.3.2 (1)  L4.8 (1) |
|  | *inhA* -770T>A + *katG* S315T | Assoc w R | 24 | L2.2.1 (16)  L4.3.3 (8) |
|  | *inhA* -777C>T + *inhA* I21V | Assoc w R | 1 | L4.1.1.3 (1) |
|  | *inhA* -777C>T | Assoc w R | 4 | L1.2.2.2 (1)  L4.1.1.3 (1)  L4.3.2 (1)  L4.4.1.1 (1) |
|  | *inhA* -777C>T + *inhA* I21T | Assoc w R | 6 | L4.3.4.1 (6) |
|  | *inhA* -777C>T + *katG* S315T | Assoc w R | 2 | L4.1.2.1 (1)  L4.3.3 (1) |
|  | inhA I21V + inhA S94A | Assoc w R- interim | 1 | L1.2.2.2 (1) |
|  | inhA S94A | Assoc w R- interim | 9 | L4.4.1.1(9) |
|  | inhA S94A + katG S315T | Assoc w R | 1 | L4.3.4.2.1 (1) |
|  | katG S315T | Assoc w R | 93 | L1.1.2 (1)  L1.2.2.2 (37)  L2.2.1 (5)  L4.1.1.1 (7)  L4.1.1.3 (11)  L4.1.2 (4)  L4.1.2.1 (1)  L4.3.2.1 (10)  L4.3.3 (2)  L4.3.4.2.1 (1)  L4.4.1.1 (9)  L4.9 (2)  L4.9.1 (2) |
| Streptomycin | *gid* 102delG | *not in the WHO catalogue | 2 | L3 (2) |
|  | *gid* 115delC | *not in the WHO catalogue | 1 | L4.4.1.1 (1) |
|  | *gid* 161_290del | *not in the WHO catalogue | 8 | L4.3.3 (8) |
|  | *gid* 351delG | *not in the WHO catalogue | 5 | L4.1.1.1 (5) |
|  | *gid* A134E | Assoc w R | 1 | L4.1.2.1 (1) |
|  | *gid* Q125* | Assoc w R | 1 | L1.1.2 (1) |
|  | *gid* L79S + rrs 514A>C | Assoc w R | 1 | L2.2.2 (1) |
|  | *rpsL* K43R + *rrs* 462C>T | Assoc w R | 1 | L2.2.1 (1) |
|  | *rpsL* K43R | Assoc w R | 10 | L4.1.1.3 (4)  L4.1.2.1 (6) |
|  | *rrs* 514A>C | Assoc w R | 2 | L4.3.3 (1)  L4.3.4.2.1 (1) |
|  | *rrs* 514A>T | *not in the WHO catalogue | 1 | L1.2.2.2 (1) |
|  | *rrs* 517C>T | Assoc w R | 35 | L1.2.2.2 (35) |
|  | *rrs* 799C>T | *not in the WHO catalogue | 1 | L4.4.1.1 (1) |
| Ethambutol | *embA* -11C>A + *embB* M306V | Assoc w R | 1 | L4.3.2.1 (1) |
|  | *embA* -16C>G + *embB* E405D | *not in the WHO catalogue | 1 | L4.1.2.1 (1) |
|  | *embA* -16C>T | *not in the WHO catalogue | 3 | L4.3.4.1 (2)  L4.3.4.2.1 (1) |
|  | *embA* -12C>T *+ embB D*354A | Assoc w R | 1 | L4.3.4.1 (1) |
|  | *embA* -29_-28delCT + *embB* Q497R | Assoc w R | 35 | L1.2.2.2 (35) |
|  | *embB* D1024N + *embB* G406A + *embB* M306I | Assoc w R | 1 | L4.1.1.3 (1) |
|  | *embB* D354A | Assoc w R | 3 | L4.3.4.1 (3) |
|  | *embB* Q497R | Assoc w R | 11 | L1.2.2.2 (2)  L4.4.1.1 (9) |
|  | *embB* Q497K | Assoc w R | 4 | L4.1.1.1 (1)  L4.4.1.1 (1)  L4.3.3 (2) |
|  | *embB* Q497K + *embB* M306I | Assoc w R | 2 | L2.2.2 (1)  L4.1.1.1 (1) |
|  | *embB* G406A + *embB* M306I + *embB* M306I + *embB* M306L + *embB* M306V | Assoc w R | 1 | L1.2.2.2 (1) |
|  | *embB* G406A | Assoc w R | 3 | L4.1.1.3 (3) |
|  | *embB* G406S | Assoc w R | 2 | L2.2.1 (1)  L4.3.2.1 (1) |
|  | *embB* M306I | Assoc w R | 12 | L2.2.1 (2)  L4.1.1.1 (2)  L4.1.2 (3)  L4.3.2.1 (2)  L4.3.3 (1)  L4.4.1.1 (2) |
|  | *embB* M306V | Assoc w R | 18 | L2.2.1 (3)  L4.1.1.1 (3)  L4.1.2.1 (2)  L4.3.3 (8)  L4.3.4.1 (1)  L4.3.4.2.1 (1) |
|  | *embB* M306L | Assoc w R | 1 | L1.1.2 (1) |
| Pyrazinamide | *pncA* -1137_*1039del |  | 1 | L4.3.2.1 (1) |
|  | *pncA* 229delC + *pncA* H57Y + *pncA* H71R + *pncA* T61P | Assoc w R | 1 | L4.4.1.1 (1) |
|  | *pncA* 375_389delCGATGAGGTCGATGT | *not in the WHO catalogue | 31 | L1.2.2.2 (31) |
|  | *pncA* 391dupG | *not in the WHO catalogue | 4 | L4.1.1.3 (4) |
|  | *pncA* 416_417dupTG | *not in the WHO catalogue | 1 | L4.3.4.2.1 (1) |
|  | *pncA* A134V | Assoc w R | 2 | L4.3.2.1 (2) |
|  | *pncA* G132A | Assoc w R | 8 | L4.3.3 (8) |
|  | *pncA* G132S | Assoc w R | 4 | L4.1.1.3 (4) |
|  | *pncA* G97C | *not in the WHO catalogue | 5 | L4.1.1.1 (5) |
|  | *pncA* G97R | Assoc w R | 1 | L1.2.2.2 (1) |
|  | *pncA* H57Y | Assoc w R | 1 | L4.4.1.1 (1) |
|  | *pncA* H71R | Assoc w R | 1 | L4.3.2.1 (1) |
|  | *pncA* H71Y | Assoc w R | 1 | L4.1.2.1 (1) |
|  | *pncA* F94L | Assoc w R | 3 | L4.4.1.1 (3) |
|  | *pncA* T135P | Assoc w R | 6 | L4.1.2.1 (6) |
|  | *pncA* T160P | Assoc w R | 3 | L4.1.2 (3) |
|  | *pncA* T61P | Assoc w R | 1 | L4.1.2.1 (1) |
|  | *pncA* T76P | Assoc w R | 2 | L4.3.2.1 (2) |
|  | *pncA* V139A | Assoc w R | 3 | L1.2.2.2 (3) |
|  | *pncA* V7A | Assoc w R- interim | 1 | L1.2.2.2 (1) |
|  | pncA V7G | Assoc w R | 1 | L4.4.1.1 (1) |
| Ethionamide | *ethA* -11A>G+ *inhA* -770T>A | Assoc w R- interim | 8 | L4.3.3 (8) |
|  | *ethA* -269_751del + *inhA* -770T>A | Assoc w R- interim | 1 | L2.2.1 (1) |
|  | *ethA* 174delC | *not in the WHO catalogue | 2 | L4.3.2.1 (2) |
|  | *ethA* 242_243insA | *not in the WHO catalogue | 4 | L1.2.2.2 (4) |
|  | *ethA* 851delC | *not in the WHO catalogue | 2 | L4.1.1 (2) |
|  | *ethA* 860delA | *not in the WHO catalogue | 6 | L4.1.2.1 (6) |
|  | *ethA* 98_336del + *inhA* S94A | *not in the WHO catalogue | 1 | L4.3.4.3.1 (1) |
|  | *ethA* A381P | *not in the WHO catalogue | 1 | L2.2.2 (1) |
|  | *ethA* T61M | *not in the WHO catalogue | 1 | L4.3.4.2.1 (1) |
|  | *ethA* G52* | *not in the WHO catalogue | 1 | L4.1.1.1 (1) |
|  | *inhA* -154G>A | Assoc w R-interim | 3 | L1.1.3.2 (1)  L1.2.2.2 (1)  L4.8 (1) |
|  | *inhA* -770T>A | Assoc w R- interim | 15 | L2.2.1 (15) |
|  | *inhA* -777C>T | Assoc w R | 7 | L1.2.2.2 (1)  L4.1.1.3 (2)  L4.1.2.1 (1)  L4.3.3 (1)  L4.3.2 (1)  L4.4.1.1 (1) |
|  | *inhA* -777C>T+ *inhA* I21T | Assoc w R | 6 | L4.3.4.1 (6) |
|  | *inhA* S94A | Assoc w R-interim | 10 | L1.2.2.2 (1)  L4.4.1.1 (8) |
| Moxifloxacin/Levofloxacin | *gyrA* A90V | Assoc w R | 27 | L1.2.2.2 (27) |
|  | *gyrA* Asp94A | Assoc w R | 2 | L4.3.3 (2) |
|  | *gyrA* Asp94G | Assoc w R | 4 | L4.1.1.1 (2)  L4.3.3 (2) |
|  | *gyrA* G88C | Assoc w R | 1 | L4.3.3 (1) |
| Amikacin/Kanamycin  /Capreomycin | *rrs* 1401A>G | Assoc w R | 2 | L4.3.3 (2) |
|  | *rrs* 1402C>A + rrs 1484G>T | Assoc w R | 2 | L4.4.1.1 (2) |
